# Supplementary material for: Space object identification via polarimetric satellite laser ranging
Source: Commun Eng. 2022 May 26;1:5. doi: 10.1038/s44172-022-00003-w (PMC10938865; doi:10.1038/s44172-022-00003-w)
Supplement: Supplementary file 1 — Supplementary Material [file 44172_2022_3_MOESM1_ESM.pdf]

Supplementary information: Space object identification via polarimetric satellite laser ranging

Nils Bartels[1], Paul Allenspacher[1], Daniel Hampf[1], Bernhard Heidenreich[2], Denise Keil[1], Ewan Schafer[1], and Wolfgang Riede[1]

[1]Deutsches Zentrum für Luft- und Raumfahrt (DLR), Institut für Technische Physik, Pfaffenwaldring 38-40, 70569 Stuttgart, Germany
[2]Deutsches Zentrum für Luft- und Raumfahrt (DLR), Institut für Bauweisen und Strukturtechnologie, Pfaffenwaldring 38-40, 70569 Stuttgart, Germany

This supplemental document is organized as follows. In Supplementary note 1 we provide a calculation of the symmetry parameters and polarization dependent intensities for the proposed retroreflector assemblies using the Mueller matrix calculus. This calculation strictly only holds at normal incidence. Thus, Supplementary note 2 and 3 provide experimental data for the symmetry parameters and far-field diffraction patterns of the retroreflector assemblies as a function of the angles of incidence. The goal is to demonstrate that the identification of a satellite is feasible, even if the orientation of CCRs in space is unknown. In Supplementary note 4 we calculate how many photons have to be acquired in a polarimetric SLR measurement in order to minimize the statistical error in the determination of symmetry parameters. Using a calculation of the SLR link budget, we find that the required photon count can be achieved even with a small and portable SLR station such as the "mini-SLR". Finally, in Supplementary note 5 we calculate the necessary range resolution that a polarimetric SLR station needs to provide in order to resolve the SLR signal coming from different CCRs mounted to a satellite.

Supplementary note 1: Calculation of the polarization properties of the retroreflector assemblies

The polarization properties of the retroreflector assemblies can be calculated using the Mueller calculus. The Mueller matrix of a waveplate ("linear retarder") $\mathbf{M}_{LR}(\theta, \delta)$ with the fast axis along θ and the retardance δ (e.g. 90° for a quarter waveplate) at normal incidence ($\phi = 0°$) is given by [1]:

$$\mathbf{M}_{LR}(\theta, \delta) = \begin{bmatrix} 1 & 0 & 0 & 0 \\ 0 & \cos^2(2\theta) + \sin^2(2\theta)\cos\delta & \sin(2\theta)\cos(2\theta)(1-\cos\delta) & -\sin(2\theta)\sin\delta \\ 0 & \sin(2\theta)\cos(2\theta)(1-\cos\delta) & \sin^2(2\theta) + \cos^2(2\theta)\cos\delta & \cos(2\theta)\sin\delta \\ 0 & \sin(2\theta)\sin\delta & -\cos(2\theta)\sin\delta & \cos\delta \end{bmatrix} \tag{S1}$$

The Mueller matrix of a polarizer $\mathbf{M}_P(\theta, \delta)$ with the polarization axis along θ is [1]:

$$\mathbf{M}_P(\theta) = \begin{bmatrix} 1 & \cos(2\theta) & \sin(2\theta) & 0 \\ \cos(2\theta) & \cos^2(2\theta) & \sin(2\theta)\cos(2\theta) & 0 \\ \sin(2\theta) & \sin(2\theta)\cos(2\theta) & \sin^2(2\theta) & 0 \\ 0 & 0 & 0 & 0 \end{bmatrix} \tag{S2}$$

Calculating the polarization properties of retroreflectors of uncoated retroreflectors generally require detailed calculations of ray paths through the retroreflector. However, it has been shown that

the Mueller matrix of CCRs with metal coated back-surfaces is approximately the Mueller matrix of an ideal mirror: [2, 3]

$$\mathbf{M}_{CCR} = \begin{bmatrix} 1 & 0 & 0 & 0 \\ 0 & 1 & 0 & 0 \\ 0 & 0 & -1 & 0 \\ 0 & 0 & 0 & -1 \end{bmatrix} \tag{S3}$$

This assumption cannot be used for uncoated retroreflectors, which operate via total internal reflection and generate different polarization states in the retroreflected beam [4].

The Mueller matrixes of the retroreflector assemblies $\mathbf{M}_{CCR,WP}$ (with a second quarter waveplate, assemblies 1-2 as defined in Tab. 1 of the main article) and $\mathbf{M}_{CCR,P}$ (with a polarizer, assemblies 3-7) are thus obtained by the following matrix multiplications:

$$\begin{aligned} \mathbf{M}_{CCR,WP} &= \mathbf{M}_{LR}\left(-\theta, 90^\circ\right)\mathbf{M}_{LR}\left(-\left(\theta+\alpha\right), 90^\circ\right)\mathbf{M}_{CCR}\mathbf{M}_{LR}\left(\theta+\alpha, 90^\circ\right)\mathbf{M}_{LR}\left(\theta, 90^\circ\right) \\ \mathbf{M}_{CCR,P} &= \mathbf{M}_{LR}\left(-\theta, 90^\circ\right)\mathbf{M}_{P}\left(-\left(\theta+\alpha\right)\right)\mathbf{M}_{CCR}\mathbf{M}_{P}\left(\theta+\alpha\right)\mathbf{M}_{LR}\left(\theta, 90^\circ\right) \end{aligned} \tag{S4}$$

Here it has been accounted for the angle α by which the polarization optics in front of the CCR (either a waveplate or a polarizer) has been rotated with respect to the outer polarization optics. Furthermore, an angle of θ on the light path towards the CCR corresponds to an angle of $-\theta$ on the way back, since the optical elements are transmitted in the opposite direction.

For the determination of the intensities I_1 und I_2 the SLR station emits light with right-circular polarization, which can be described with the Stokes vector $(1, 0, 0, 1)^\tau$, where the symbol τ is used to indicate the transposed vector. Similarly, the intensities I_3 and I_4 are measured with left-circular polarized light with the Stokes vector $(1, 0, 0, -1)^\tau$. Before detection, the light that is reflected from the retroreflector assembly passes the PSA which consists of a quarter waveplate (with $\theta = +45^\circ$ or $\theta = -45^\circ$ for right-circular and left-circular detection, respectively) and a polarizer. The detected intensities are then given by the first element $\hat{n}_1$ of the Stokes vector for the overall light paths.

$$\begin{aligned} I_1 &= \mathbf{M}_P(0^\circ)\mathbf{M}_{LR}(+45^\circ, 90^\circ)\mathbf{M}_{CCR,WP/P}(\theta, \alpha)(1, 0, 0, 1)^\tau \hat{n}_1 \\ I_2 &= \mathbf{M}_P(0^\circ)\mathbf{M}_{LR}(-45^\circ, 90^\circ)\mathbf{M}_{CCR,WP/P}(\theta, \alpha)(1, 0, 0, 1)^\tau \hat{n}_1 \\ I_3 &= \mathbf{M}_P(0^\circ)\mathbf{M}_{LR}(+45^\circ, 90^\circ)\mathbf{M}_{CCR,WP/P}(\theta, \alpha)(1, 0, 0, -1)^\tau \hat{n}_1 \\ I_4 &= \mathbf{M}_P(0^\circ)\mathbf{M}_{LR}(-45^\circ, 90^\circ)\mathbf{M}_{CCR,WP/P}(\theta, \alpha)(1, 0, 0, -1)^\tau \hat{n}_1 \end{aligned} \tag{S5}$$

By inserting Eqs. S1 to S5 into Eq. 2, the following results for the symmetry parameters are obtained for the assemblies with two quarter wave plates (assemblies 1-2 of Tab. 1)

$$P_1 = 0, \qquad P_2 = -P_3 = \cos(4\alpha) \tag{S6}$$

and for assemblies with a quarter wave plate and a polarizer (assemblies 3-7 of Tab. 1)

$$P_1 = \sin(2\alpha), \qquad P_2 = P_3 = -\sin(2\alpha) \tag{S7}$$

This means that the symmetry parameters are properties that are at least to a first approximation independent of the orientation θ of the retroreflector array. Instead, the parameters only depend on the type of polarization optics (quarter wave plate versus polarizer) and the angle α, which can be fixed during the manufacturing of the retroreflector assembly. This is why they can be used to identify the satellite. Supplementary Fig. 1 shows the calculated intensities and symmetry parameters of the retroreflector assemblies defined in Tab. 1.

Supplementary note 2: Dependence of symmetry parameters of incidence angles

The calculation of the intensities and symmetry parameters presented in Supplementary note 1 strictly only holds at normal incidence ($\phi = 0^\circ$) and for an ideal retroreflector and ideal polarization optics.

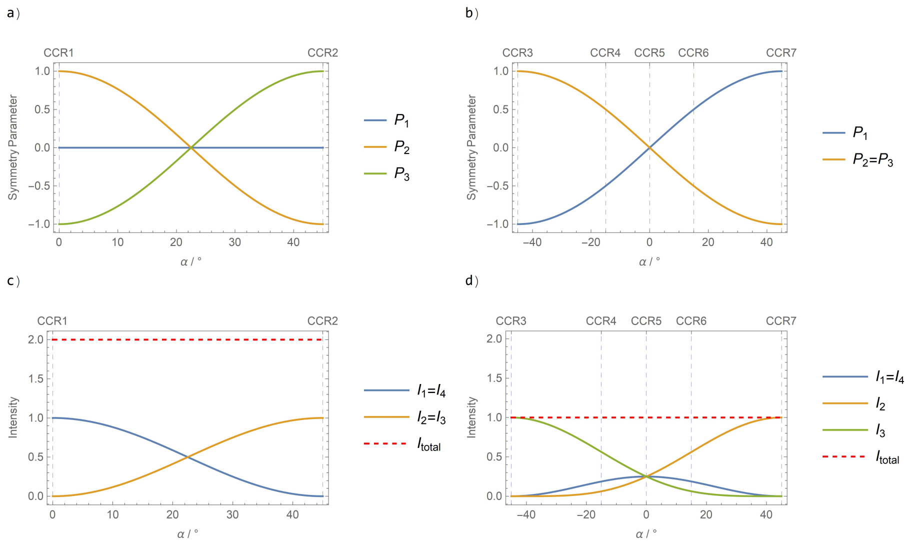

Supplementary Figure 1: Calculated symmetry parameters and intensities. a Symmetry paramters of retroreflector assemblies with two quarter waveplates. The retroreflector assemblies are defined in Tab. 1. The angle α is characteristic for each assembly. **b** Intensities of retroreflector assemblies with two quarter waveplates. **c** Symmetry paramters of retroreflector assemblies with a quarter waveplate and a polarizer. **d** Intensities of retroreflector assemblies with a quarter waveplate and a polarizer.

Although we have chosen optical components that are known to have a small angular dependence of their polarization properties, the polarization properties of the true-zero order waveplate[5] the retroreflector [6, 7, 4] and the wire-grid polarizer[8] will all depend on the incidence angles θ_i and ϕ. Although this behavior could be treated by theoretical modeling (e.g. via polarization ray tracing [9, 10]), this is fairly complicated, since there are 6 possible different ray paths through the retroreflector (meaning the sequence in which the ray is reflected from the three reflecting back-facets of the CCR). Furthermore, it is necessary to calculate the (polarization-dependent) far-field diffraction pattern, ideally considering additional effects such as manufacturing errors.[4] This has only recently been achieved by introducing the complex amplitude of a retroreflecting beam into a simplified Kirchhoff diffraction equation.[6] While such a modelling is beyond the scope of this work, it would clearly be very useful to optimize the retroreflector assemblies (e.g. the choice of the metal coating on the back facets of the CCR) to achieve the same symmetry parameters over a wide range of incidence angles and within the FFPD. In this work, we instead decided to assess the angular dependence experimentally, by measuring the intensities (integrated power) I_1 to I_4 over a vast range of incidence angles and for all 7 suggested retroreflector assemblies. We used a commercial polarimeter (Thorlabs, PAX1000IR1) to assist in the alignment of the polarimetric setup.

Supplementary Fig. 2 shows the measured intensities of assembly 1, which is a CCR with two quarter waveplates mounted with $\alpha = 0°$. In this case, most of the intensity is obtained for I_1 and I_4, which corresponds to the emission and detection of right, and left-circular polarized light, respectively. As opposed to this, there is a very small intensity in the cross-polarization channels I_2 and I_3.

The intensities in Supplementary Fig. 2 can then be used to evaluate the symmetry parameters P_1 to P_3 (see Supplementary Fig. 3). Ideally, the assembly should have the symmetry parameters $P_1 = 0$, $P_2 = 1$ and $P_3 = -1$. We find that the symmetry parameter P_1 is approximately 0 (error less than 10%) for all incidence angles. As opposed to this, the symmetry parameters P_2 and P_3 vary with incidence angles, especially for $\phi > 30°$. A very similar behavior was observed for the symmetry parameters of CCR assembly 2 (data not shown). We thus introduced only two assemblies with two

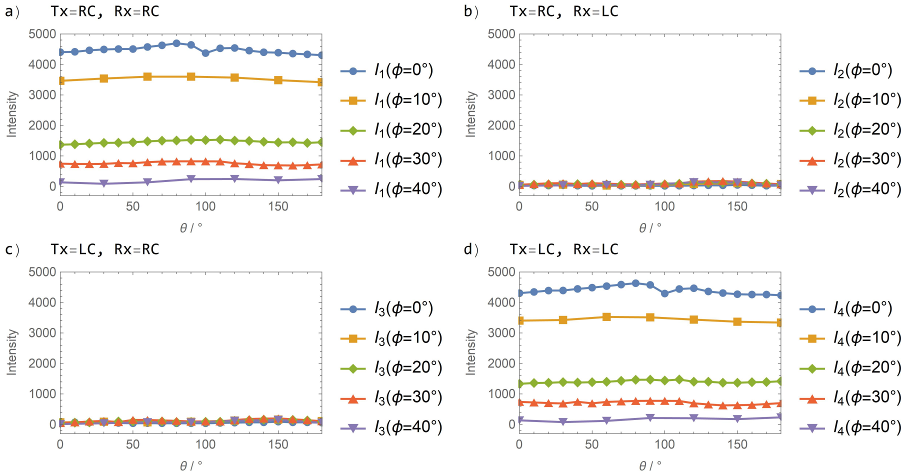

Supplementary Figure 2: Polarization-dependent intensities for CCR assembly 1. **a** Intensities measured with different incidence angles ϕ (as given in the figure legend) as a function of the rotation angle θ. The emitter (Tx) was set to emit photons with right-circular (RC) polarization and photons with RC polarization were detected. **b** Same as panel a), but with RC emission and left-circular (LC) detection. **c** Same as panel a), but with LC emission and RC detection. **d** Same as panel a), but with LC emission and LC detection.

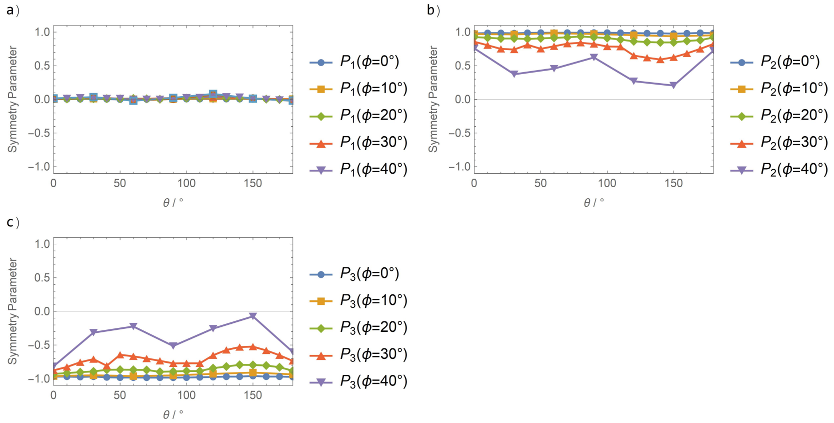

Supplementary Figure 3: Symmetry parameters of CCR assembly 1. **a** Symmetry parameter P_1 derived from the intensities plotted in Supplementary Fig. 2 at different incidence angles ϕ (as given in the figure legend) as a function of the rotation angle θ. **b** Symmetry parameter P_2.

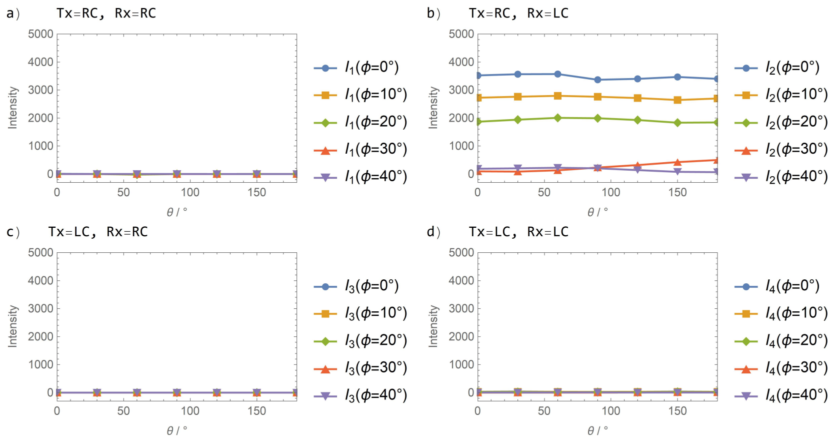

Supplementary Figure 4: Polarization-dependent intensities for CCR assembly 7. **a** Intensities measured with different incidence angles ϕ (as given in the figure legend) as a function of the rotation angle θ. The emitter (Tx) was set to emit photons with right-circular (RC) polarization and photons with RC polarization were detected. **b** Same as panel a), but with RC emission and left-circular (LC) detection. **c** Same as panel a), but with LC emission and RC detection. **d** Same as panel a), but with LC emission and LC detection.

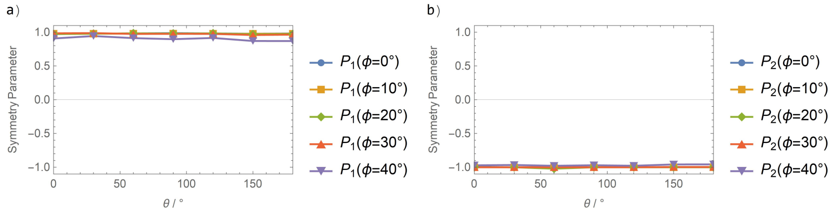

Supplementary Figure 5: Symmetry parameters of CCR assembly 7. **a** Symmetry parameter P_1 derived from the intensities plotted in Supplementary Fig. 4 at different incidence angles ϕ (as given in the figure legend) as a function of the rotation angle θ. **b** Symmetry parameter P_2. **c** Symmetry parameter P_3.

quarter waveplates (assembly 1 and assembly 2) and suggest that these assemblies should only be used at incidence angles of $\phi \leq 30°$. This restriction can, for example, be achieved by recessing the front face of the CCR. The maximum and minimum values obtained within this range of $\phi \leq 30°$ (and for any value of θ_i) have been plotted as error bars for CCR assemblies 1 and 2 in Supplementary Fig. 5. Note that the reflectivity of the CCR is very low at higher incidence angles anyway.

As opposed to retroreflector assemblies 1 and 2, we found that the assemblies 3 to 7 only have a very small dependence of the symmetry parameters on the angles of incidence. As an example, Supplementary Fig. 4 shows the measured intensities for CCR assembly 7. For this assembly almost all of the intensity is in the I_2 channel, where RC polarized light is emitted and LC polarized light is detected. The angular dependence of the symmetry parameters is provided in Supplementary Fig. 5.

By also performing similar measurements for the other CCR assemblies with polarizer, we have again used the maximum and minimum values of the symmetry parameters to derive the error bars given in Fig. 5. For these assemblies we included the data for $\phi = 40°$ (which is the maximum angle which we can measure in the experimental setup) for the error bars.

We would like to note in passing that we also measured the symmetry parameters of the CCR without any polarization optics. The data is not shown here, but is very similar to the data of CCR assembly 1 as provided in Supplementary Fig. 2 and 3. We thus conclude that most of the non-ideal behavior in

the polarization properties in assemblies 1 and 2 is introduced by the CCR itself. The polarizer in the CCR assemblies 3 to 7 reduces the angular dependence of the symmetry parameters, since it defines a specific axis of linear polarization for light upon entering the CCR.

Supplementary note 3: Additional data for the measurement of far-field diffraction patterns

In Sec. 3 of the main article, we provided data for the diffraction patterns and calculated symmetry parameters for CCR assembly 6, which is an assembly with polarizer, at an angle of incidence of $\phi = 30°$. In Supplementary Fig. 6 to 11, we provide additional data for assembly 6 at $\phi = 0°$ and for assembly 2 at $\phi = 30°$ and $\phi = 0°$. The data shows that there is only a small variation of the symmetry parameters (typically in the range of $\Delta P = \pm 0.1$) within the relevant range of diffraction angles (between 21 and 53 µrad) relevant due to the velocity aberration of a satellite in low Earth orbit.

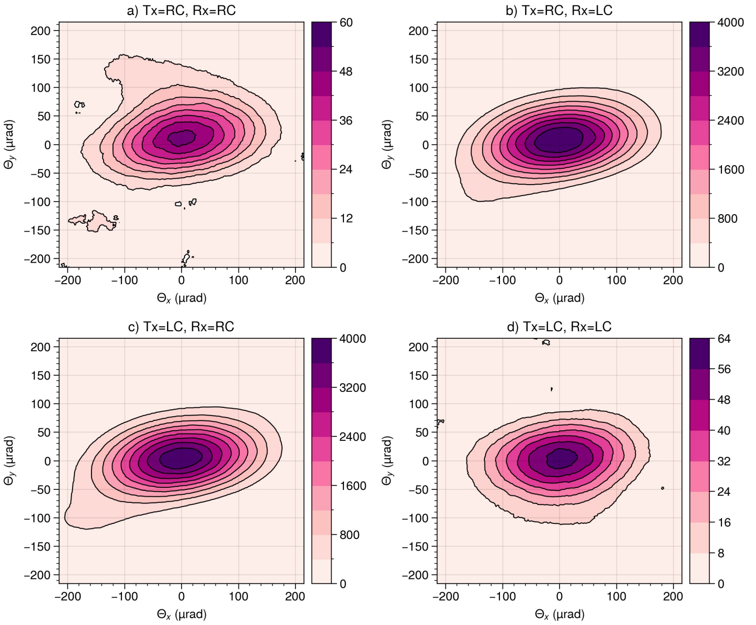

Supplementary Figure 6: Polarization dependent far-field diffraction pattern (FFDP) of retroreflector assembly 2. **a** FFDP measured at an incidence angle of $\phi = 30°$ when irradiated with right-circular polarization (Tx=RC) and detected with right-circular polarization (Rx=RC). **b** Same as panel a, but for Tx=RC and Rx=LC. **c** Same as panel a, but for Tx=LC and Rx=RC. **d** Same as panel a, but for Tx=LC and Rx=LC.

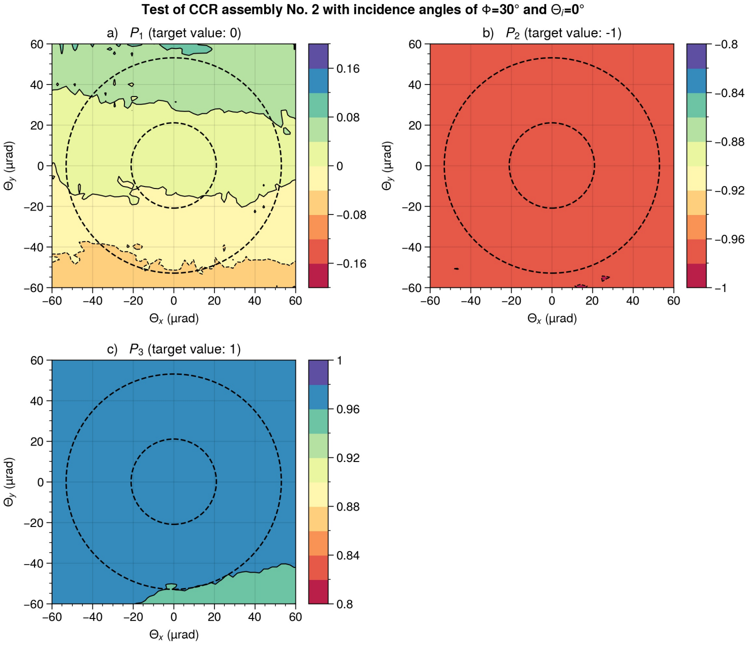

Supplementary Figure 7: Symmetry parameters of CCR assembly 2. **a** Symmetry parameter P_1 as a function of the diffraction angles Θ_x and Θ_y at an incidence angle of $\phi = 30°$. The dashed circles indicate the minimum (inner circle) and the maximum (outer circle) velocity aberration. **b** Same as panel a), but the symmetry parameter P_2. **c** Same as panel a), but the symmetry parameter P_3.

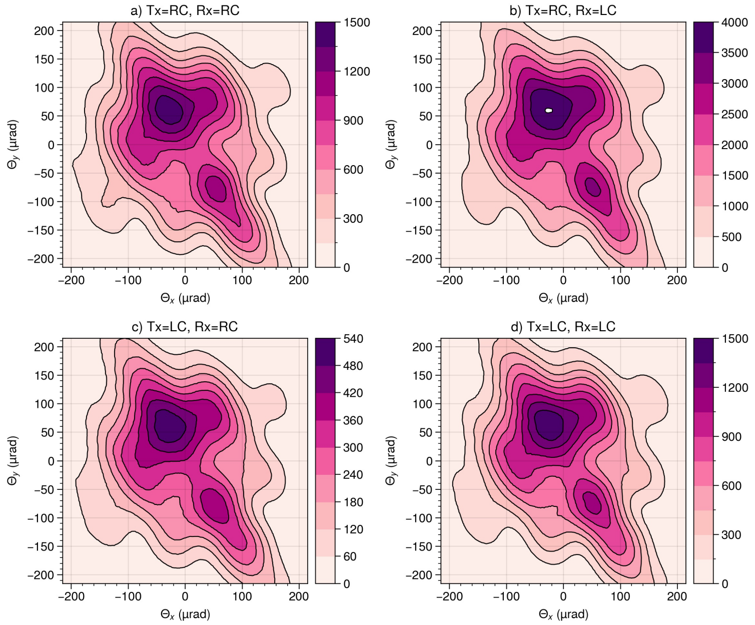

Supplementary Figure 8: Polarization dependent far-field diffraction pattern (FFDP) of retroreflector assembly 6. **a** FFDP measured at an incidence angle of $\phi = 0°$ when irradiated with right-circular polarization (Tx=RC) and detected with right-circular polarization (Rx=RC). **b** Same as panel a, but for Tx=RC and Rx=LC. **c** Same as panel a, but for Tx=LC and Rx=RC. **d** Same as panel a, but for Tx=LC and Rx=LC.

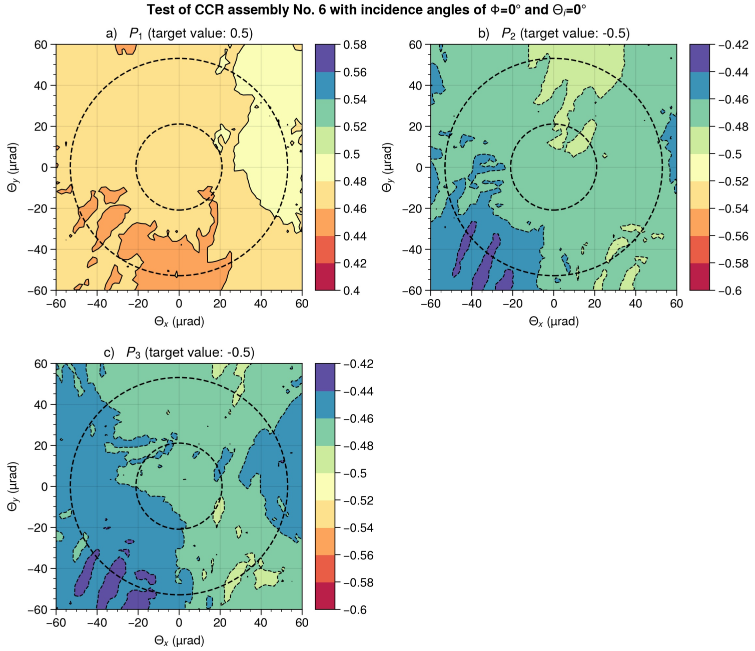

Supplementary Figure 9: Symmetry parameters of CCR assembly 6. **a** Symmetry parameter P_1 as a function of the diffraction angles Θ_x and Θ_y at an incidence angle of $\phi = 0°$. The dashed circles indicate the minimum (inner circle) and the maximum (outer circle) velocity aberration. **b** Same as panel a), but the symmetry parameter P_2. **c** Same as panel a), but the symmetry parameter P_3.

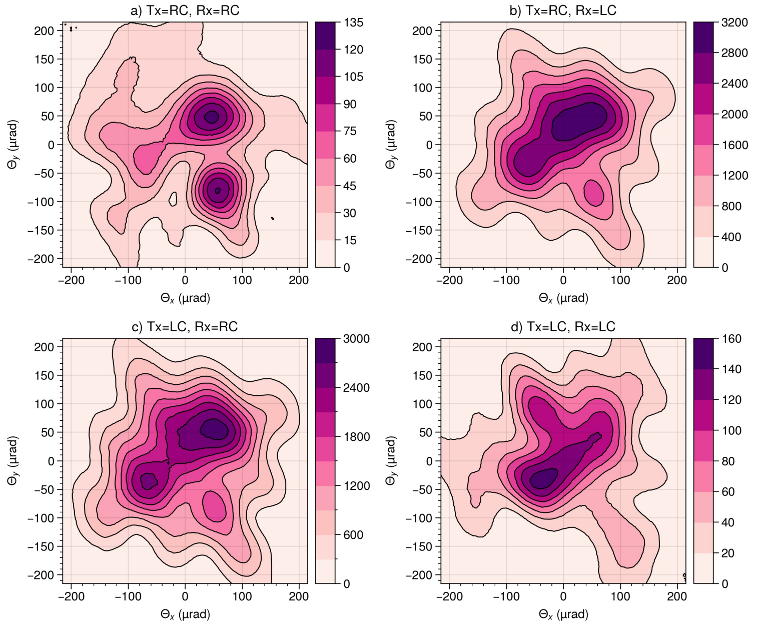

Supplementary Figure 10: Polarization dependent far-field diffraction pattern (FFDP) of retroreflector assembly 2. **a** FFDP measured at an incidence angle of $\phi = 0°$ when irradiated with right-circular polarization (Tx=RC) and detected with right-circular polarization (Rx=RC). **b** Same as panel a, but for Tx=RC and Rx=LC. **c** Same as panel a, but for Tx=LC and Rx=RC. **d** Same as panel a, but for Tx=LC and Rx=LC.

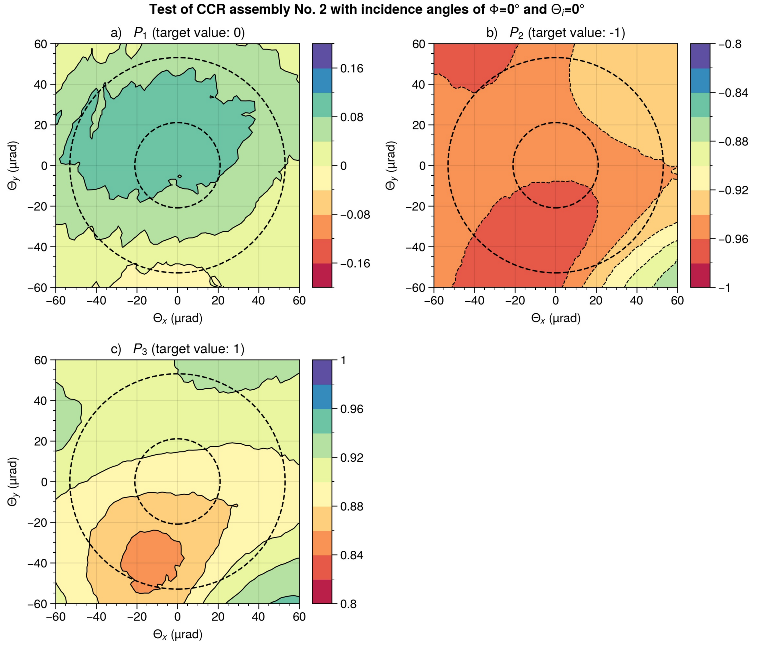

Supplementary Figure 11: Symmetry parameters of CCR assembly 2. **a** Symmetry parameter P_1 as a function of the diffraction angles Θ_x and Θ_y at an incidence angle of $\phi = 0°$. The dashed circles indicate the minimum (inner circle) and the maximum (outer circle) velocity aberration. **b** Same as panel a), but the symmetry parameter P_2. **c** Same as panel a), but the symmetry parameter P_3.

Supplementary note 4: Statistical error in the determination of the symmetry parameters and polarimetric SLR link budget

In this supplementary note, we will first derive how many photons need to be detected to achieve an accurate determination of the symmetry parameters and thus allow for a reliable identification of the space object. We will then calculate the link budget for SLR of a satellite equipped with a small CCR (half inch diameter front face, as typically used for small satellites in LEO) and assess whether the required number of photons can be obtained with our SLR station “mini-SLR”.

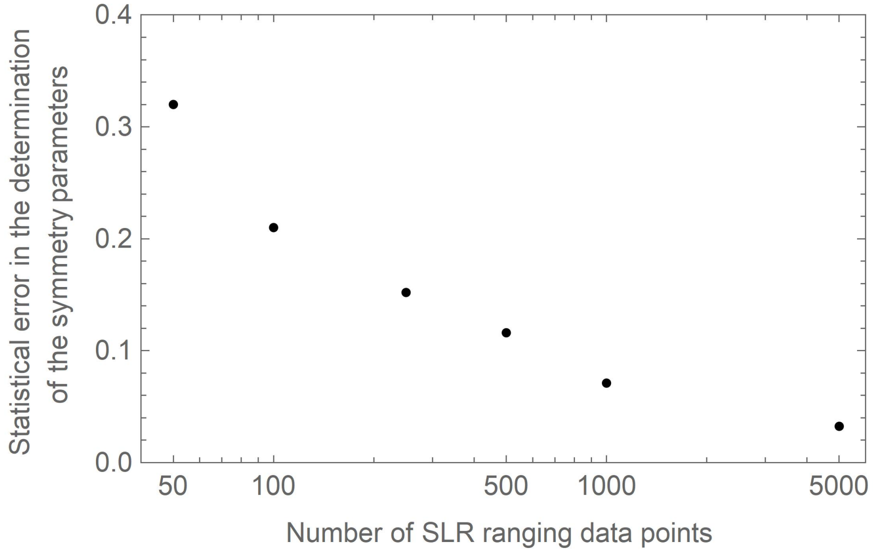

Supplementary Figure 12: Statistical error of symmetry parameters. The calculated, statistical error of the symmetry parameter is plotted as a function of the number of valid SLR ranging data points (detected photons).

To assess the required number of detected photons we simulated SLR data (100 data sets with a photon count between 50 and 5000) for a retroreflector assembly with expectation values for the symmetry parameters of $P_1 = P_2 = P_3 = 0$ (meaning identical intensities for I_1 to I_4 for a large number of detected photons) with a calculation written in Wolfram Mathematica. The calculation is available to the reader and the link is provided in the code availability statement of the main manuscript. The basic idea is that we use a random integer number generator to build a data set of n photons with the attribute of being detected during τ_1, τ_2, τ_3 or τ_4, which is represented by an integer (1,2,3 or 4). To calculate the intensities I_1 to I_4 for our data sets (generated with the random number generator), we simply count the number of elements 1,2,3 and 4. Then we calculate the symmetry parameters P_1 to P_3 via Eq. 2 of our main manuscript. For each number of photons, the generation of a data set and the calculation of symmetry parameters was repeated for 100 times. The “Statistical error in the determination of the symmetry parameters” (plotted Supplementary Fig. 12) is defined as the difference between the largest and the smallest derived symmetry parameter (from these 100 datasets) divided by 2. We find that 1000 detected photons (labeled as “Number of SLR ranging data points” in Supplementary Fig. 12) will generate a statistical error of less than ± 0.07 for the determination of the symmetry parameter. This value is thus our targeted minimum number of detected photons. It should be noted, that this consideration assumes that all photo-electrons used for the evaluation of the symmetry parameters represent photons that returned from the retroreflectors in space to the SLR station. In reality, there are sources of "noise" such as back-scattering from clouds or a dark count

rate of the detector. Such effects have to be minimized (e.g. via an optical filter that blocks sunlight and transmits the laser wavelength). Furthermore, time intervals with a low count rate should be excluded from the data evaluation. When the intensities for I_1 to I_4 would be no signals, this case also corresponds to the assembly 5 case in terms of the orthogonality. In order to minimize the statistical error of the evaluated symmetry parameters, it is furthermore important that the detection takes place over many switching circles for the different polarization states. We estimate that the total integration time for the measurement of each polarization state should be at least 3 seconds in order to average out scintillation due to atmospheric turbulence, which can spawn a frequency range up to kHz [13]. This will cut-off intensity fluctuations with a frequency higher that 1Hz.

The number of detected photons in an SLR experiment can be estimated with the radar link equation.[12] According to this equation, the number of detected photo electrons per sent laser pulse η_p during satellite laser ranging is given by

$$\eta_P = \frac{E_T \lambda}{hc} \eta_t \frac{G_T}{4\pi d^2} \frac{\sigma_0}{4\pi d^2} \frac{\sigma}{\sigma_0} A_r \eta_r \eta_q T_a^2 T_c^2 \tag{S8}$$

and depends on the laser pulse energy (E_T), the laser wavelength (λ), the transmitter gain (G_T), the distance from the SLR station to the satellite (d), the retroreflector optical cross section at normal incidence (σ_0), a correction factor that accounts for the CCR cross section as function of incidence angle and velocity aberration (σ/σ_0), the area of the receiving telescope (A_r), the efficiency of the receiving optics (η_r), the efficiency of the photon detector (η_q) and the atmospheric and cirrus-cloud transmissions (T_a and T_c). In this formula it is assumed, that the SLR station points the emitted laser beam to the position that the satellite will reach after the photon travel time (point-ahead principle[14]). Furthermore, it is assumed, that effects of atmospheric turbulence on the photon link budget can be neglected. For the mini-SLR, the laser beam divergence is 50 μrad and thus smaller than the turbulence at good seeing (typically between 5 and 10 μrad [16]).

For polarimetric satellite laser ranging at a high repetition rate, additional effects reducing the photon count have to be considered in the photon link budget:

$$\eta_{p,pol} = \eta_p \eta_{pol} \eta_{PC} \eta_{LCVR}. \tag{S9}$$

In Eq. S9, η_{pol} is a polarimetric transmission factor, that accounts for photons that are rejected either at the polarizer of the polarization state analyzer (PSA) of the SLR station or at the (wire-grid) polarizer that is part of CCR assemblies 3-7. This transmission factor takes the value 0.5 for CCR assemblies no. 1-2 (retroreflector assemblies without polarizer) and 0.25 for the CCR assemblies 3-7 (retroreflector assemblies with polarizer). To illustrate this, we would like to refer to Supplementary Fig. 1, which shows the intensities I_1 to I_4 calculated according to Eq. S5. From Supplementary Fig. 1c) at $\alpha = 0°$ (which corresponds to CCR assembly 1), we find that the detected intensities are $I_1 = I_4 = 1$ and $I_2 = I_3 = 0$. If there were no polarization optics on the CCR assembly and no PSA in the SLR station, these intensities would be $I_1 = I_2 = I_3 = I_4 = 1$. This means that on average there is a 50% loss of transmission for assembly 1 and thus $\eta_{pol} = 0.5$. As opposed to this, for CCR assembly 3 (Supplementary Fig. 1d at $\alpha = 0°$), we have $I_3 = 1$ and $I_1 = I_2 = I_4 = 0$. This means that $\eta_{pol} = 0.25$.

The term η_{pc} considers that SLR with a high laser pulse repetition rate requires strategies to avoid the detection of photons from atmospheric back-reflections due to the up-going laser beam. For some SLR stations, this is achieved via burst mode ranging, leading to a reduced number of detected pulses.[17] For the mini-SLR, burst mode ranging is not necessary, since the laser emitter and the receive telescope are separated by a distance of approximately 1 m. Due to this separation, the field of view of the detector (75 μrad half angle) and the laser beam (with a beam divergence of 50 μrad) will only start to overlap at a distance d larger than 6 km. Since the irradiance I of a (point) source follows an inverse square law with the distance $I \propto 1/d^2$, this is sufficient to effectively suppress the detection of atmospheric back-reflections due to Rayleigh scattering and $\eta_{PC} = 1$.

Finally, it also needs to be considered that the liquid crystal variable retarders (LCVRs) used in the PSG and PSA require time to switch between left-circular and right-circular polarization states.

This switching time depends on the exact type of LCVR, its temperature and the wavelengths range of operation. Typically, the rise time (increasing retardance) τ_{rise} of the LCVR is slower (e.g. 50 ms at 1064 nm) than the fall time τ_{fall} (below 1 ms). During the switching time, the polarization states set at the PSA and PSG are not well-defined and thus the detected photons cannot be used for evaluating the symmetry parameters. If the polarization on the emitter is switched as RC→LC→LC→RC and on the receiver as RC→RC→LC→LC and if the switching for RC→LC occurs with τ_{rise} and from LC→RC with τ_{fall}, a sequence of switching of polarization states (the suggested sequence corresponds to $\tau_1 \to \tau_3 \to \tau_4 \to \tau_2$) requires two slow polarization changes and two fast polarization changes. Thus, the correction factor for the switching time η_{LCVR} is given by

$$\eta_{LCVR} = 1 - \frac{2\tau_{rise} + 2\tau_{fall}}{\tau_1 + \tau_2 + \tau_3 + \tau_4}. \tag{S10}$$

and gives a value of $\eta_{LCVR} = 0.49$ for $\tau_{rise} = 50$ ms, τ_{fall}=1 ms and $\tau_1 = \tau_2 = \tau_3 = \tau_4 = 50$ ms. We would like to note in passing, that the LCVR on the PSA should be switched with a delay (the two-way photon travel time τ_{travel}) compared to the LCVR of the PSG.

As an example, Supplementary Table 1 provides the relevant parameters for the SLR station "mini-SLR" (after recent upgrades) and for a satellite with a half inch diameter CCR.

An important parameter for the link budget is the correction factor (σ/σ_0) that accounts for the CCR cross section as a function of incidence angle and velocity aberration. We have calculated this for the retroreflector with 6.1 mm radius of the front face, a length of 10.16 mm, a refractive index of 1.4496 (fused silica) and a wavelengths of 1064 nm for different incidence angles ϕ using the equations given in the appendix of Ref. [14]. We find that σ/σ_0 is always larger than 0.05, if ϕ is smaller than 30° over the relevant range of velocity aberrations (see Supplementary Fig. 13).

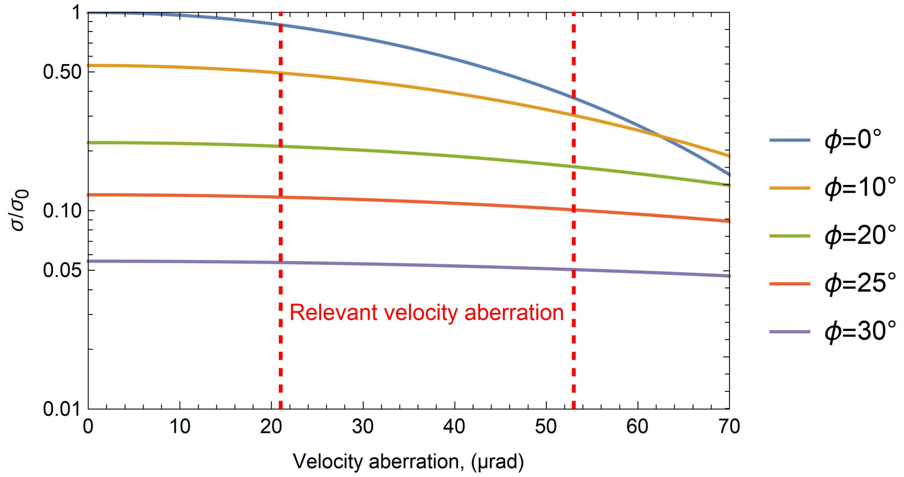

Supplementary Figure 13: Retroreflector cross section. Normalised retroreflector optical cross section, σ/σ_0, as a function of velocity aberration, for a range of retroreflector incidence angles ϕ as calulated for a retroreflector with a 6.1 mm radius of the front face, a length of 10.16 mm, a refractive index of 1.4496 (fused silica) and a wavelengths of 1064 nm. The minimum and maximum values for the velocity aberration are given as red dashed vertical lines.

The distance d between the satellite and the SLR ground station depends on the orbit of the satellite. Assuming a satellite with a circular orbit at a height of $h_S = 600$ km and neglecting the station height above sea level, the distance d is correlated to the zenith angle ϕ_Z via

$$d = \sqrt{R_e^2 \cos^2\phi_Z + 2R_e h_S + h_S^2} - R_e \cos\phi_Z, \tag{S11}$$

Supplementary Table 1: Input data for calculating the example polarimetric SLR link budget

Paramter	Value	Information
E_T	150 µJ	Laser pulse energy
λ	1064 nm	Wavelength
η_t	0.5	Efficiency of transmitter
G_T	$\frac{8}{\theta_t^2} \exp\left[-2\left(\frac{\theta}{\theta_t}\right)^2\right]$ $= 1.9 \cdot 10^9$ / rad^2	Transmitter gain for a Gaussion beam, where θ_t is the divergence half angle between the beam center and the $1/\exp(1)^2$ intensity point (estimated to 50µrad) and where θ is the beam pointing error estimated to half the beam divergence ($\frac{\theta}{\theta_t} = 0.5$)
σ_0	$\varrho \frac{4\pi}{\lambda^2}\left(\pi r_{CC}^2\right)$ $= 1.36 \cdot 10^5$ m^2	CCR cross section at normal incidence and without velocity aberration, with $\varrho = 0.9$ (reflectivity of retroreflector) and $r_{CC} = 6.1$ mm (radius of retroreflector aperture)
σ/σ_0	0.05	Correction factor accounting for the CCR cross section as function of incidence angle ϕ and velocity aberration.[14, 11] The value of 0.05 corresponds to an incidence angle of $\phi \approx 30°$ (see Supplementary Fig. 13).
A_r	$\frac{\pi}{4}\left(d_1^2 - d_2^2\right)$ $= 0.034$m^2	Area of the receiving telescope, where $d_1 = 0.23$ m is the diameter of the primary telescope mirror, which is partially shaded by the secondary mirror with the diameter $d_2 = 0.1$ m.
η_r	0.5	efficiency of receiving optics
η_q	0.3	efficiency of detector
T_a	0.8	atmospheric transmission (estimated)
T_c	0.8	cirrus cloud transmission (estimated)
η_{pol}	0.5/0.25	polarimetric transmission factor: 0.5 for assemblies 1 and 2 (assemblies with two wave plates) and 0.25 for assemblies 3-7 (assemblies with a wave plate and a polarizer)
η_{PC}	1	transmission factor for SLR systems with burst mode pulse collision avoidance
η_{LCVR}	0.49	transmission factor accounting for the switching time of LCVRs

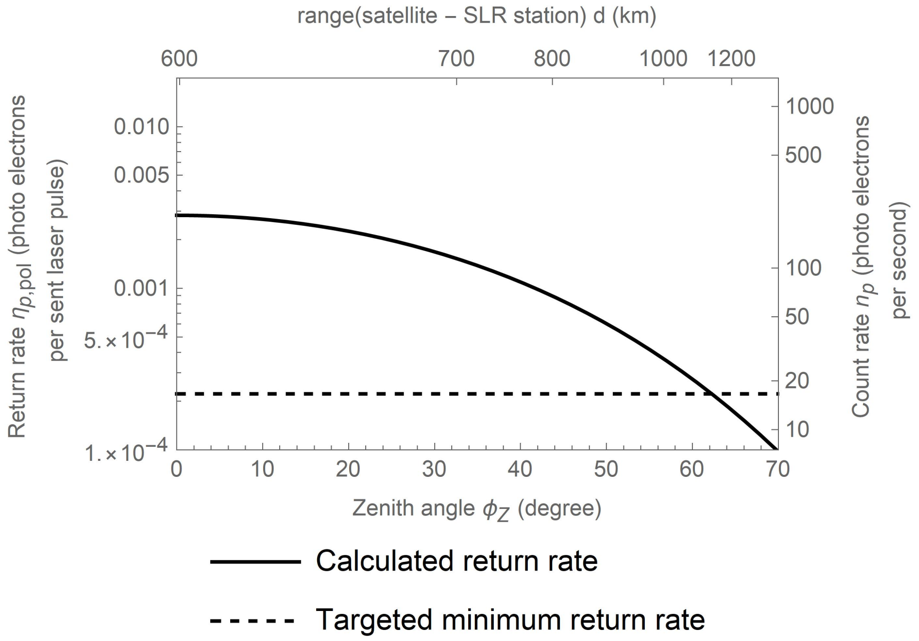

Supplementary Figure 14: Return rate. Calculated return rate $\eta_{p,pol}$ as a function of the zenith angle ϕ of the satellite over the SLR station. The calculation uses performance parameters of the SLR station "mini-SLR" and a half inch diameter retroreflector with η_{pol}=0.25. The red dashed line describes the targeted minimum return rate which has to be maintained over 60 seconds.

where R_e is the radius of the Earth.

Under these assumptions, Supplementary Fig. 14 shows the calculated return rate $\eta_{p,pol}$ as a function of the zenith angle ϕ of the satellite over the SLR station. The count rate $n_{p,pol}$ (number of detected photoelectrons per second) is plotted on the right y-axis and is obtained by multiplication of $\eta_{p,pol}$ with the laser pulse repetition rate (75 kHz in case of the mini-SLR).

Satellites in a low Earth orbit typically pass over the SLR within several minutes. However, we define that we want to be able to obtain the necessary photon count of 1000 photons within 60 seconds, since the SLR measurement will need time to lock its tracking to the satellite before the polarimetric measurement can start and because seeing might not always be optimal. This means that we optimally require a photon count higher than 17 photons per second (1000 photons/(60 s)),which corresponds to a return rate of 0.02 % at 75 kHz. This "targeted minimum return rate" is indicated as a dashed line in Supplementary Fig. 14 and is well below the calculated return rate for zenith angles smaller than 60 degrees. This means that the count rate of the "mini-SLR" should be sufficiently large for the identification of the satellite, even though most other SLR stations use much larger telescopes (the "mini-SLR" is designed to be small and portable). We would like to note in passing, that the return rate of the polarimetric SLR experiment should be maintained below 10% to avoid multi-photon returns. Such multi-photon returns would only be detected as a single photon by the a single-photon avalanche diode (SPAD) detector and would thus lead to a bias in the intensity and thus the symmetry parameters. By keeping the photon detection rate below 10% (if necessary, the laser power can easily be reduced with neutral density filters), it is ensured that that the probability for the arrival of 2 photons (where the second photon would not be detected) is maintained below 1%.

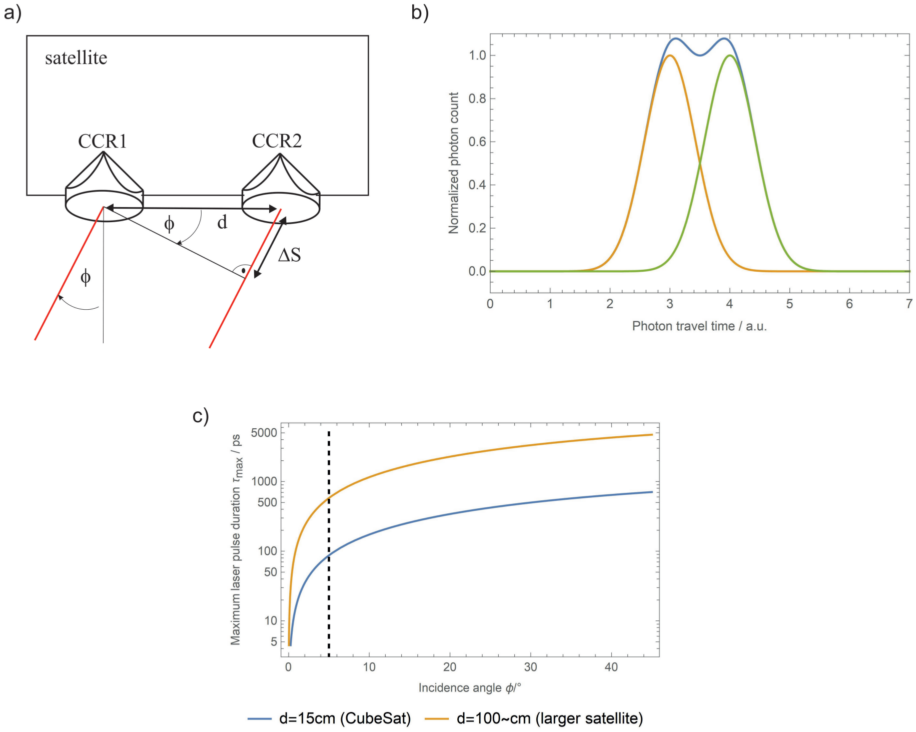

Supplementary Figure 15: Range resolution. **a** Sketch of the geometry used to calculate the path difference ΔS between the signal of two retroreflectors (labeled as CCR1 and CCR2) mounted to a satellite, when irradiated at an incidence angle ϕ. **b** The SLR signal (photon count versus photon travel time) originating from two CCRs (yellow and green lines) can be resolved by fitting the combined signal (blue line) with two functions. **c** Maximum pulse duration of laser pulses from the SLR station required to resolve the signal of two CCRs as a function of the incidence angle ϕ and for two different distances d between the CCRs. The blue line corresponds to d=15 cm (realistic for a 2 unit cube satellite) and the yellow line to d=100 cm (for a larger satellite).

Supplementary note 5: Calculation of the required SLR range resolution to distinguish different CCR signals

In case the satellite uses more than one retroreflector (ID with more than one digit), the signal coming from these retroreflector assemblies has to be resolved in time to identify the satellite.

As can be seen from Supplementary Fig. 15a), the path difference ΔS for a laser beam incident on two CCRs mounted to the wall of the satellite depends on the incidence angle ϕ via

$$\Delta S = d \sin \phi, \tag{S12}$$

where d is the distance between the CCRs. A photon that is retroreflected from CCR2 will then have a range delay Δt_{Ph} (compared to a photon reflected from CCR1) of

$$\Delta t_{Ph} = 2\frac{\Delta S}{c} = 2\frac{d \sin \phi}{c}, \tag{S13}$$

where c is the speed of light and the factor of 2 accounts for that the laser path difference in the bidirectional SLR experiment is twice the distance ΔS. The photon signal returning from the different CCRs has to be separated by fitting the photo-electron signal with two functions (one for each CCR)

that take the form of the temporal profile of the emitted laser pulses (see Supplementary Fig. 15b). We assume that these functions can be easily resolved, if the laser pulse duration τ (the full width at half maximum of the temporal profile of the laser pulse) is smaller than the time difference between the signals.

Thus, the maximum pulse duration τ_{max} is given by

$$\tau_{max} = 2\frac{d\sin\phi}{c}. \qquad \text{(S14)}$$

Fig. 15c) shows the maximum pulse duration τ_{max} needed to resolve the CCRs as a function of the incidence angle ϕ for distances between the retroreflectors of $d = 15$ cm (realistic for a 16 U CubeSat) and $d = 100$ cm (realistic for a larger satellite) as blue and yellow lines, respectively. If we require that we want to able to resolve the signal at incidence angles of ϕ greater than 5°, the larger satellite ($d = 100$ cm) can be ranged with a maximum pulse duration of 600 picoseconds. Thus, the identification can easily be achieved with the SLR station "miniSLR", which uses a laser with 450 ps pulse duration. As opposed to this, CCRs on the CubeSat ($d = 15$ cm) cannot be resolved with the mini-SLR, which is optimized for being small and portable. However, many other SLR stations use lasers with a pulse duration as low as 10 ps (most SLR stations provide information in the single-shot precision on the ILRS website[15]), which is easily sufficient to resolve the CCRs even under these circumstances. SLR typically uses a gated detector, which will only be active for a short time before and after the expected arrival time of retroreflected photons for each laser pulse. The goal of this gating is the reduction of noise and to avoid a signal degradation due to the dead time of the single-photon detector.[17] It is important that the active time of the range gate is sufficiently long to detect the signal from all retroreflectors mounted to the satellite.

References

[1] R. A. Chipman, Handbook of optics, Chapter 22: Polarimetry, *Optical Society of America* (1995).

[2] S. E. Segre & V. Zanza, Mueller calculus of polarization change in the cube-corner retroreflector, *J. Opt. Soc. Am. A* **20**, 1804-1811 (2003).

[3] W. He et al., Polarization properties of a cornercube retroreflector with three-dimensional polarization ray-tracing calculus, *Appl. Opt.* **52**, 4527–4535 (2013).

[4] T. W. Murphy & S. D. Goodrow, Polarization and far-field diffraction patterns of total internal reflection corner cubes, *Appl. Opt.* **52**, 117-126 (2013).

[5] Meadowlark optics, Sources of Error in Retarders and Waveplates, *Application note* (2005). `https://www.meadowlark.com/store/PDFs/Retarders.pdf`

[6] Z. Hui & L. Song & Z. Wenhao & C. Yuwei, Far-field diffraction pattern of a nonideal retroreflector for polarized light with an oblique incidence, *Appl. Opt.* **59**, 2621-2631 (2005).

[7] R. Kalibjian, Polarization preserving corner cubes, *Optics & Laser Technology* **44**, 239-246 (2012).

[8] X. J. Yu & H. S. Kwok, Optical wire-grid polarizers at oblique angles of incidence, *J. Appl. Phys.* **93**, 4407-4412 (2003).

[9] G. Yun & K. Crabtree & R. A. Chipman, Three-dimensional polarization ray-tracing calculus I: definition and diattenuation, *Appl. Opt.* **50**, 2855-2865 (2011).

[10] G. Yun & S. C. McClain & R. A. Chipman, Three-dimensional polarization ray-tracing calculus II: retardance, *Appl. Opt.* **50**, 2866-2874 (2011).

[11] N. Bartels et al., Design and qualification of a recessed satellite cornercube retroreflector for ground-based attitude verification via satellite laser ranging, *CEAS Space Journal* **11**, 391-403 (2019).

[12] J. J. Degnan, Millimeter Accuracy Satellite Laser Ranging: a Review, *Contributions of Space Geodesy to Geodynamics: Technology* **25** (1993).

[13] P. G. Goetz et al., Modulating Retro-reflector Lasercom Systems at the Naval Research Laboratory, *Conference: MILITARY COMMUNICATIONS CONFERENCE, 2010 - MILCOM 2010* (2010).

[14] P. C. Stephenson, Satellite laser ranging photon-budget calculations for a single satellite cornercube retroreflector: Attitude control tolerance, *Tech. rep., Australian Government, Department of Defence, National Security and ISR Division, DST-Group-TR-3172* (2015).

[15] Website of the international laser ranging service (accessed March 26th, 2021).`https://ilrs.gsfc.nasa.gov/network/system_performance/global_report_cards/perf_2019q4_wLLR.html`

[16] A. Abahamid et al., Seeing, outer scale of optical turbulence, and coherence outer scale at different astronomical sites using instruments on meteorological balloons, *A&A* **412**, 1123-1127 (2004).

[17] D. Hampf et al., Satellite laser ranging at 100 kHz pulse repetition rate, *CEAS Space J.* **11**, 363-370 (2019).
